# Missense variants in the TNFA epitopes and their effects on interaction with therapeutic antibodies—in silico analysis

**DOI:** 10.1186/s43141-021-00288-y

**Published:** 2022-01-10

**Authors:** Tamim Ahsan, Abu Ashfaqur Sajib

**Affiliations:** 1 Molecular Biotechnology Division, National Institute of Biotechnology, Dhaka, 1349 Bangladesh; 2 Department of Genetic Engineering & Biotechnology, University of Dhaka, Dhaka, 1000 Bangladesh

**Keywords:** TNFA, Anti-TNFA antibody, Therapeutic antibody, Genetic variants, Autoimmune disease

## Abstract

**Background:**

Tumor necrosis factor alpha (TNFA) is an important cytokine that influences multiple biological processes. It is one of the key mediators of acute and chronic systemic inflammatory reactions and plays a central role in several autoimmune diseases. A number of approved monoclonal antibodies (mAbs) are widely used to subside these autoimmune diseases. However, there is a high rate of non-responsiveness to treatments with these mAbs. Therefore, it is important to be able to predict responses of the patients in an individualistic manner to these therapeutic antibodies before administration. In the present study, we used in silico tools to explore the effects of missense variants in the respective epitopes of four therapeutic anti-TNFA mAbs—adalimumab (ADA), certolizumab pegol (CZP), golimumab (GLM), and infliximab (IFX)—on their interactions with TNFA.

**Results:**

The binding affinities of CZP and ADA to corresponding epitopes appear to be reduced by four (TNFA^R131Q^, TNFA^E135G^, TNFA^R138Q^, and TNFA^R138W^) and two (TNFA^G66C^ and TNFA^G66S^) variants, respectively. The binding of GLM and IFX appears to be affected by TNFA^R141S^ and TNFA^R138W^, respectively. TNFA^G66C^ and TNFA^G66S^ may be associated with autoimmune diseases, whereas TNFA^E135G^, TNFA^R138W^, and TNFA^R141S^ may be pathogenic per se.

**Conclusion:**

These variants may contribute to the observed inter-individual variability in response to anti-TNFA mAbs treatments and be used as markers to predict responses, and thus optimize therapeutic benefits to the patients.

**Supplementary Information:**

The online version contains supplementary material available at 10.1186/s43141-021-00288-y.

## Background

Tumor necrosis factor alpha (TNFA) is a cytokine that mediates pleotropic influences on a myriad of cellular processes including cell proliferation and differentiation, immune responses, apoptosis, necroptosis, etc [1, 2]. It is primarily produced as a type II transmembrane protein in stable homotrimeric arrangements, which is proteolytically cleaved into soluble cytokine (sTNF) by TNFA-converting enzyme (TACE) [3]. Its pleotropic actions are mediated through interaction with two distinct receptors—TNF receptor type 1 (TNFR1) and TNF receptor type 2 (TNFR2) [4]. TNFR1, which is expressed in nearly all cells, is the major high-affinity TNFA receptor [5, 6]. TNFR1-TNFA interaction primarily activates nuclear factor kappa B (NF-κB) and mitogen-activated protein kinase (MAPK) pathways and leads to proinflammatory responses, apoptosis, and necroptosis [1, 7]. TNFR2 is expressed in CD4^+^ and CD8^+^ T lymphocytes, endothelial cells, microglia, oligodendrocytes, neurons, cardiac myocytes, thymocytes, and human mesenchymal stem cells [6]. TNFR2-TNFA interaction activates both classical and alternative NFκB pathways [7] and causes cell survival, cell expansion, angiogenesis, etc. [8, 9]. In addition to working independently, TNFR1 and TNFR2 can also engage in complex cross-talks to maintain a delicate balance between cell survival and apoptosis [10].

TNFA is one of the key mediators of acute and chronic systemic inflammatory reactions. TNFA induces its own secretion as well as the production of other inflammatory cytokines and chemokines, and thus plays a central role in several autoimmune diseases such as rheumatoid arthritis (RA), inflammatory bowel disease (IBD) including Crohn’s disease (CD), ulcerative colitis (UC), multiple sclerosis (MS), systemic lupus erythematosus (SLE), systemic sclerosis, psoriasis (PS), psoriatic arthritis (PsA), and ankylosing spondylitis (AS) [11–13]. Modulation of inflammation by antibody-based inhibitors of receptor-TNFA interactions leads to dramatic improvements in patients suffering from these autoimmune diseases [14]. Several FDA-approved anti-TNFA monoclonal antibodies (mAbs) such as adalimumab (ADA), certolizumab pegol (CZP), golimumab (GLM), and infliximab (IFX) are commercially available and widely used to treat such diseases [15].

ADA is a fully human anti-TNFA mAb [16], whereas CZP is composed of a humanized F_ab_ fragment (50 kDa) of IgG1 isotype conjugated to a 40-kDa polyethylene glycol (PEG) moiety [17]. GLM is a human IgG1 mAb, which was originally produced in transgenic mice immunized with human TNFA, but is commercially produced in a recombinant cell line [18]. IFX is a chimeric monoclonal IgG1 antibody with human constant and murine variable regions [19]. ADA, CZP, GLM, and IFX bind to sites on TNFA that overlap with its sites of interaction with the receptor [20–23].

ADA, CZP, GLM, and IFX are widely prescribed to treat RA, CD, UC, PS, PsA, and AS. In fact, ADA and IFX are among the top-selling therapeutic mAbs [24]. These protein-based therapeutics are quite expensive [25, 26]. However, their clinical efficacy is sometimes limited due to a high rate (30–40%) of non-responsiveness [27, 28]. Hence, it is important to be able to predict responses of the patients in an individualistic manner to these therapeutic antibodies before administering them. To the best of our knowledge, no single-nucleotide polymorphism (SNP) or variant in TNFA epitopes has been associated with responses to corresponding mAbs. In this study, we investigated the interactions of ADA, CZP, GLM, and IFX mAbs with their respective epitopic variants in TNFA using in silico tools.

## Methods

### Missense variants in the epitopes for anti-TNFA monoclonal antibodies

The amino acid residues of TNFA that constitute the epitopes for ADA, CZP, GLM, and IFX were retrieved through literature search [20–23]. Missense variants within the TNFA epitopes were obtained using the Ensembl Genome Browser [29].

### Prediction of the effects of missense variants on TNFA-mAb interactions

X-ray crystallographic structures of TNFA-ADA (PDB ID: 3WD5), TNFA-CZP (PDB ID: 5WUX), TNFA-GLM (PDB ID: 5YOY), and TNFA-IFX (PDB ID: 4G3Y) complexes were retrieved from the Protein Data Bank (PDB) [30]. Since 3WD5 and 4G3Y contained a single light and a heavy chain of the antibody with a single epitope, these complexes were not customized any further. 5WUX and 5YOY contain complex structures with multiple epitopes and heavy and light chains, and therefore required customization for analysis. H (CZP heavy chain), L (CZP light chain), and E (TNFA molecule) chains from TNFA-CZP, and I (GLM heavy chain), F (GLM light chain), and A (TNFA molecule) chains from TNFA-GLM X-ray crystallographic structures were retained for further analysis. The rest of the chains were removed using UCSF Chimera 1.14 [31] to retain interactions between a single antibody molecule and a single TNFA protomer. These four structures of antigen-antibody complexes were used as inputs to predict the effects of the epitopic missense variants on interaction with the relevant antibodies using mCSM-PPI2 [32], SAAMBE-3D [33], and MutaBind [34].

### 3D modeling and stabilizing energy calculation of TNFA variant-mAb complexes

The amino acid sequence of TNFA (UniProt accession number: P01375) was retrieved from UniProt [35]. Deletion of the signal peptide (first 76 residues) and substitution of residues at the variant sites was performed manually. Amino acid sequences of the heavy and light chains of the mAbs were obtained from the Therapeutic Structural Antibody Database (Thera-SAbDab) [36]. 3D models of TNFA-mAb complexes were generated using template-based modeling in SWISS-MODEL [37]. X-ray crystallographic structures of the four TNFA-mAb complexes (previously mentioned) were used as templates. Chains in the models were renamed (wherever applicable) to H (mAb heavy chain), L (mAb light chain), and A (TNFA molecule) using UCSF Chimera 1.14 [31]. Stabilizing energies of TNFA-mAb complexes were calculated using the PPCheck web server [38].

### Analyses of structures, interfaces, and interactions

Changes in the TNFA structure due to the variants were predicted using Missense3D [39]. 2D maps of interactions between TNFA and H and L chains of mAbs were generated with iCn3D using the default parameters [40]. Distances between selected atoms and areas of interacting surfaces between TNFA and mAb chains were measured using UCSF Chimera 1.14 [31] and iCn3D [40], respectively. The number of bonds and interactions (van der Waals interactions, H-bonds, weak H-bonds, and ionic interactions) between TNFA and mAb chains was calculated with Arpeggio—a web server for calculating interatomic interactions in protein structures [41]. Antigen-antibody interfaces were visualized using PyMOL [42]. In all cases, 3D models of TNFA-mAb complexes were used as the inputs.

### Prediction of the variants’ effects on TNFA-receptor interactions

To assess whether the variants predicted to significantly affect the TNFA-mAb interactions would also affect interactions between TNFA and its receptors, X-ray crystallographic structure of the TNFA-TNFR2 complex (PDB ID: 3ALQ) was retrieved from the PDB [30]. The PDB file contained multiple TNFA and TNFR2 chains. Therefore, the structure was customized by keeping only chain A and removing all the other chains using UCSF Chimera 1.14 [31]. TNFA-TNFR2 interactions were checked using iCn3D [40]. Only two chains (R and T) of TNFR2 were found to form bonds with the selected TNFA chain. So, these two chains were retained and the other chains were deleted. The PDB file thus prepared was used as an input in mCSM-PPI2 [32], SAAMBE-3D [33], and MutaBind2 [43] to assess the effects of selected missense variants on TNFA-TNFR2 interactions. We could not retrieve any X-ray crystallographic structure of TNFA-TNFR1 complex.

### Assessment of pathogenicity of the variants

DisGeNET [44] and PhenoScanner V2 [45] databases were searched for diseases associated with the selected variants. PolyPhen2- and SIFT-predicted pathogenicity-associated information of these selected variants were retrieved from Ensembl Genome Browser [29]. Pathogenicity was also predicted using PMut [46], Meta-SNP [47], and PredictSNP 1.0 [48].

## Results

### Missense variants in the epitopes of anti-TNFA monoclonal antibodies

Amino acid residues that constitute the epitopes of TNFA for ADA [20], CZP [21], GLM [22], and IFX [23] were retrieved through literature survey (Table 1). Based on the data collected via Ensembl, eleven missense variants were found in each of the epitopes for ADA and CZP (Tables 2 and 3). Seven and ten missense variants were found in the epitopes for GLM and IFX, respectively (Tables 4 and 5).

**Table 1:** Epitopes of TNFA for adalimumab, certolizumab pegol, golimumab and infliximab

Antibody	TNFA epitopes	References
Adalimumab	Pro-19, Gln-20, Glu-23, Lys-65 To Gln-67, Glu-10 TO Pro-113, Tyr-141, Ala-145, Glu-146, Thr-71, His-72, Thr-77, Thr-79, Ser-81, Lys-89 To Asn-91, Glu-135 To Asn-137	[20]
Certolizumab	Gly-24, Asp-45, Gln-47, Thr-77, Ile-83, Val-85, Ser-86, Gln-88, Thr-89, Lys-90, Arg-131, Glu-135, Asn-137, Arg-138, Pro-139, Asp-140	[21]
Golimumab	Gly-24, Lys-65, Gln-67, Ser-71, Glu-104, Thr-105, Pro-106, Glu-107, Gly-108, Ala-111, Arg-138, Asp-140, Tyr-141	[22]
Infliximab	Gln-67, Pro-70, Ser-71, His-73, Gln-102, Thr-105, Glu-107, Ala-109, Glu-110, Arg-138, Asn-137, Tyr-141	[24]

**Table 2:** Effects of epitopic missense variants on TNFA-adalimumab interaction

SNP_ID	Amino acid position^**a**^	Amino acid change	∆∆G (Kcal/mol)^**b**^			Antibody chains	MutaBind^**b**^	PPcheck
			mCSM-PPI2	SAAMBE-3D	MutaBind		∆∆G (kcal/mol)	Total stabilizing energy (kJ/mol)
WT	–	–	–	–	–	H	–	− 58.31
	–	–	–	–	–	L	–	− 222.88
rs1454071630	66	G>C	1.345	0.38	1.94	H	1.65	− 58.09
	–	–	–	–	–	L	2.33	− 208.55
rs1454071630	66	G>S	1.279	0.45	2	H	1.6	− 58.05
	–	–	–	–	–	L	2.38	− 207.41
rs552363141	72	T>N	0.116	0.15	0.12	H	0.7	− 58.33
	–	–	–	–	–	L	0.46	− 222.89
rs946653352	72	T>P	0.107	0.19	0.64	H	1.35	− 58.32
	–	–	–	–	–	L	1.23	− 222.89
rs758652888	73	H>Q	0.373	0.36	0.13	H	0.9	− 58.33
	–	–	–	–	–	L	0.63	− 222.93
rs753130938	73	H>R	0.101	0.25	0.21	H	0.94	− 58.33
	–	–	–	–	–	L	0.66	− 222.93
rs1473587140	110	E>K	0.17	1.64	0.86	H	1.23	− 58.13
	–	–	–	–	–	L	1.19	− 225.92
rs760036765	111	A>T	–0.272	0.21	0.97	H	0.96	− 58.25
	–	–	–	–	–	L	1.16	− 225.75
rs777545571	135	E>G	0.235	0.59	0.48	H	1.22	− 58.53
	–	–	–	–	–	L	1.03	− 229.29
rs758189183	135	E>K	0.048	0.55	0.3	H	1.03	− 58.47
	–	–	–	–	–	L	0.84	− 227.42
rs763621932	141	Y>S	0.28	1.07	1.02	H	1.16	− 58.3
	–	–	–	–	–	L	1.3	− 217.37

^a^Amino acid positions were calculated excluding the signal peptide (76 amino acids)

^b^Positive values of ∆∆G indicate decreasing affinity

**Table 3:** Effects of epitopic missense variants on TNFA-certolizumab pegol interaction

SNP_ID	Amino acid position^**a**^	Amino acid change	∆∆G (Kcal/mol)^**b**^			Antibody chains	MutaBind^**b**^	PPcheck
			mCSM-PPI2	SAAMBE-3D	MutaBind		∆∆G (kcal/mol)	Total stabilizing energy (kJ/mol)
WT	–	–	–	–	–	H	–	− 269.69
	–	–	–	–	–	L	–	− 152.18
rs1298153213	45	D>N	− 0.619	0.95	0.61	H	0.98	− 277.93
	–	–	–	–	–	L	0.46	− 152.11
rs373646181	85	V>F	− 1.58	0.01	0.7	H	0.97	− 271.68
	–	–	–	–	–	L	0.54	− 152.18
rs778731236	85	V>G	0.416	1.55	0.99	H	1.08	− 266.54
	–	–	–	–	–	L	0.47	− 152.18
rs373646181	85	V>I	0.339	0.53	0.79	H	1.01	− 274.09
	–	–	–	–	–	L	0.48	− 152.17
rs376368223	131	R>Q	− 0.078	0.94	2.31	H	2.52	− 236.86
	–	–	–	–	–	L	0.57	− 152.06
rs777545571	135	E>G	0.784	0.67	2.25	H	1.03	− 269.49
	–	–	–	–	–	L	1.98	− 145.12
rs758189183	135	E>K	1.119	0.67	1.86	H	0.92	− 279.21
	–	–	–	–	–	L	1.74	− 157.25
rs770509340	138	R>Q	0.026	2.25	1.77	H	0.41	− 269.36
	–	–	–	–	–	L	1.96	− 120.82
rs141307820	138	R>W	0.652	1.53	2.19	H	0.51	− 269.49
	–	–	–	–	–	L	2.42	− 142.18
rs370893734	140	D>N	0.64	0.75	0.64	H	0.42	− 269.56
	–	–	–	–	–	L	0.88	− 147.77
rs370893734	140	D>Y	− 0.122	0.25	0.92	H	0.74	− 269.58
	–	–	–	–	–	L	1.21	− 149.7

^a^Amino acid positions were calculated excluding the signal peptide (76 amino acids)

^b^Positive values of ∆∆G indicate decreasing affinity

**Table 4:** Effects of epitopic missense variants on TNFA-golimumab interaction

SNP_ID	Amino acid position^**a**^	Amino acid change	∆∆G (Kcal/mol)^**b**^			Antibody chains	MutaBind^**b**^	PPcheck
			mCSM-PPI2	SAAMBE-3D	MutaBind		∆∆G (kcal/mol)	Total stabilizing energy (kJ/mol)
WT	–	–	–	–	–	H	–	− 290.62
	–	–	–	–	–	L	–	− 55.79
rs140654183	105	T/N	0.463	0.67	0.84	H	0.58	− 294.29
	–	–	–	–	–	L	0.98	− 56.15
rs760036765	111	A/T	− 0.711	0.54	1.35	H	1.62	− 300.47
	–	–	–	–	–	L	1.01	− 55.71
rs770509340	138	R/Q	0.075	1.47	0.49	H	0.25	− 284.33
	–	–	–	–	–	L	0.96	− 54.37
rs141307820	138	R/W	0.668	1.03	0.72	H	0.44	− 285.08
	–	–	–	–	–	L	1.23	− 55.81
rs370893734	140	D/N	1.279	0.96	2.4	H	0.75	− 293.72
	–	–	–	–	–	L	1.41	− 62.05
rs370893734	140	D/Y	0.213	0.78	4	H	1.01	− 285.94
	–	–	–	–	–	L	1.42	− 53.07
rs763621932	141	Y/S	2.219	1.34	2.45	H	2.07	− 270.91
	–	–	–	–	–	L	1.43	− 55.23

^a^Amino acid positions were calculated excluding the signal peptide (76 amino acids)

^b^Positive values of ∆∆G indicate decreasing affinity

**Table 5:** Effects of epitopic missense variants on TNFA-infliximab interaction

SNP_ID	Amino acid position^**a**^	Amino acid change	∆∆G (Kcal/mol)^**b**^			Antibody chains	MutaBind^**b**^	PPcheck
			mCSM-PPI2	SAAMBE-3D	MutaBind		∆∆G (kcal/mol)	Total stabilizing energy (kJ/mol)
WT	–	–	–	–	–	H	–	− 188.42
	–	–	–	–	–	L	–	− 148.55
rs1427914441	70	P/S	0.669	1.05	0.6	H	1.18	− 180.87
	–	–	–	–	–	L	0.31	− 148.18
rs758652888	73	H/Q	0.264	1.03	0.18	H	0.47	− 188.4
	–	–	–	–	–	L	1.41	− 137.66
rs753130938	73	H/R	0.034	0.84	0.31	H	0.53	− 188.38
	–	–	–	–	–	L	1.45	− 145.3
rs140654183	105	T/N	0.315	0.62	− 0.1	H	1.09	− 186.46
	–	–	–	–	–	L	0.66	− 148.51
rs1473587140	110	E/K	0.491	1	0.17	H	1.18	− 184.14
	–	–	–	–	–	L	0.63	− 148.55
rs770509340	138	R/Q	0.875	1.81	1.2	H	0.72	− 186.64
	–	–	–	–	–	L	1.56	− 146.21
rs141307820	138	R/W	1.234	1.01	1.2	H	1.01	− 186.21
	–	–	–	–	–	L	1.58	− 153.47
rs370893734	140	D/N	1.439	1.25	0.66	H	0.93	− 156.05
	–	–	–	–	–	L	0.89	− 149.23
rs370893734	140	D/Y	0.398	0.6	2.03	H	1.28	− 160.89
	–	–	–	–	–	L	1.29	− 158.06
rs763621932	141	Y/S	0.72	0.86	0.57	H	1.3	− 180.87
	–	–	–	–	–	L	1.02	− 148.78

^a^Amino acid positions were calculated excluding the signal peptide (76 amino acids)

^b^Positive values of ∆∆G indicate decreasing affinity

### Binding affinities of TNFA epitopic variants to anti-TNFA therapeutic antibodies

The effects of the identified missense variants on TNFA-mAb interactions were predicted in two different ways. First, changes in the binding affinity (∆∆G = ∆G_mutant_ − ∆G_wild-type_) were predicted using the crystallographic structures with mCSM-PPI2 [32], SAAMBE-3D [33], and MutaBind [34]. Second, the 3D models of TNFA-mAb complexes (both wild-type and epitopic variants of TNFA) were generated and total stabilizing energies of the complexes were determined using PPCheck [38]. In all cases, ∆∆G values of > 1.0 kcal/mol (4.18 kJ/mol) were considered to be a significant reduction in binding affinity [49]. In case of antigen-antibody interactions, |∆∆G| > 1.0 kcal/mol may be considered as an indication of significant changes in antibody binding affinity [50].

None of the variants was predicted by every tool to reduce the binding affinity of ADA to TNFA by more than 1.0 kcal/mol (Table 2). Only the mCSM-PPI2 and MutaBind predicted values of ∆∆G due for TNFA^G66C^ and TNFA^G66S^ (rs1454071630) variants were > 1.0 kcal/mol. Since ∆∆G values predicted by SAAMBE-3D were very close to 0.53 kcal/mol [33] in both cases, TNFA^G66C^ and TNFA^G66S^ might actually destabilize the TNFA-ADA complex. PPcheck predicted interactions between these epitopic variants of TNFA with the L chain of ADA were weaker as well. Although MutaBind predicted weaker interactions of these variants with both L and H chains of ADA, binding to the L chain was more severely affected (∆∆G > 2.3 kcal/mol) than that of the H chain (∆∆G ≈ 1.6 kcal/mol).

Epitopes with TNFA^R131Q^ (rs376368223), TNFA^E135G^ (rs777545571), TNFA^R138Q^ (rs770509340), and TNFA^R138W^ (rs141307820) variants were predicted to have weaker interactions with CZP, although none of the variants was predicted to significantly decrease (∆∆G > 1.0 kcal/mol) by all the tools (Table 3). Although mCSM-PPI2, SAAMBE-3D, and MutaBind predicted destabilizing effect of TNFA^E135k^ (rs758189183) on interaction with CZP, this finding was not corroborated with the 3D model. TNFA^R131Q^ weakened the interactions between TNFA and the H chain of CZP. On the other hand, TNFA^E135G^, TNFA^R138Q^, and TNFA^R138W^ weakened interactions between TNFA and the L chain of CZP.

Significant reduction in affinity (∆∆G > 1.0 kcal/mol) of GLM for its TNFA epitope was convincingly predicted to be caused due to the TNFA^Y141S^ (rs763621932) variant by all the tools used in this study (Table 4). Both MutaBind and PPCheck predicted weaker interactions between TNFA^Y141S^ and the H chain of GLM.

mCSM-PPI2, SAAMBE-3D, and MutaBind predicted significantly weakened interaction (∆∆G > 1.0 kcal/mol) between TNFA^R138W^ (rs141307820) variant and IFX (Table 5). But the effect could not be replicated with 3D modeling.

### Effects of epitopic variants on TNFA structure and interaction with anti-TNFA antibodies and TNFR2

**Fig. 1:**
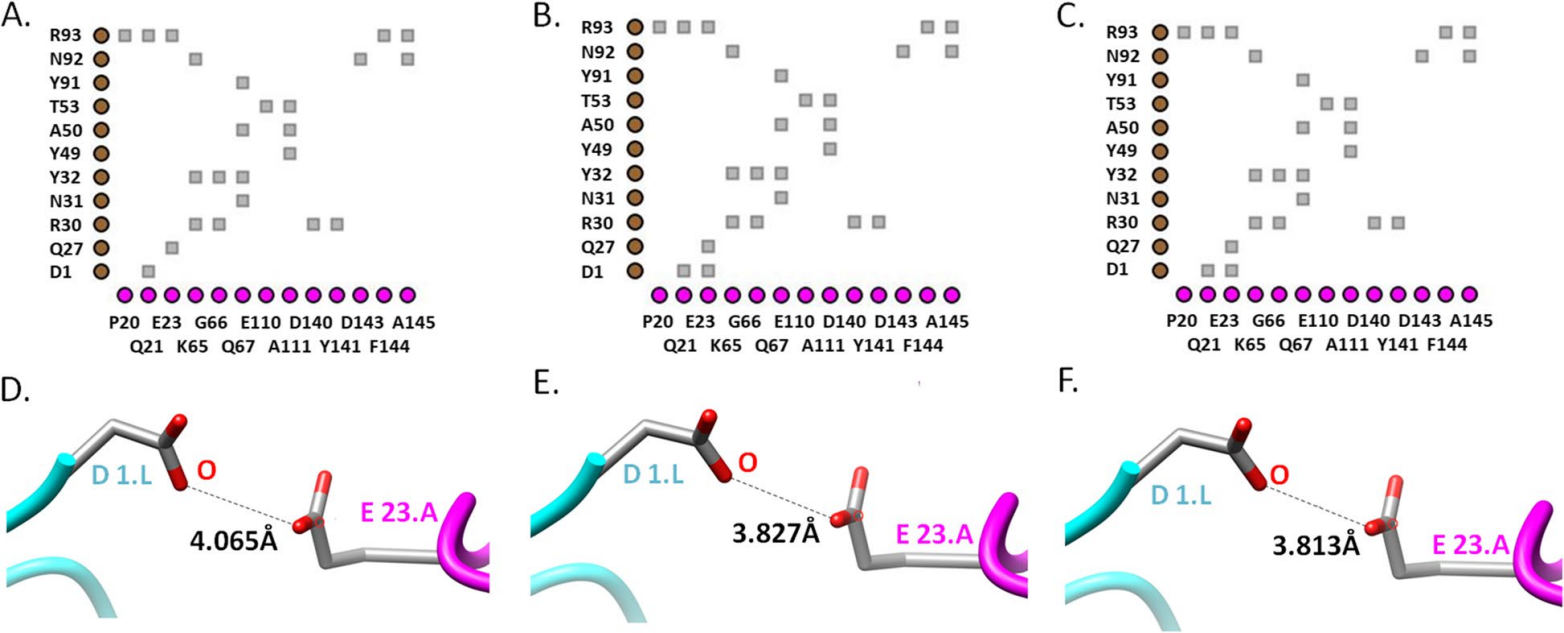
Interactions between TNFA and adalimumab. 2D plots of interactions between the light chain of adalimumab and TNFA^WT^ (**A**), TNFA^G66C^ (**B**), and TNFA^G66S^ (**C**) structures. Distance between TNFA^Glu-23^ and ADA^Asp-1^ in the L chain in wild-type (**D**), TNFA^G66C^, (**E**) and TNFA^G66S^ (**F**) variant structures. In the 2D interaction plots TNFA residues are shown along the horizontal axis and adalimumab light chain residues are delineated along the vertical axis. Grey- contacts/interactions (within 4 Å)

**Fig. 2:**
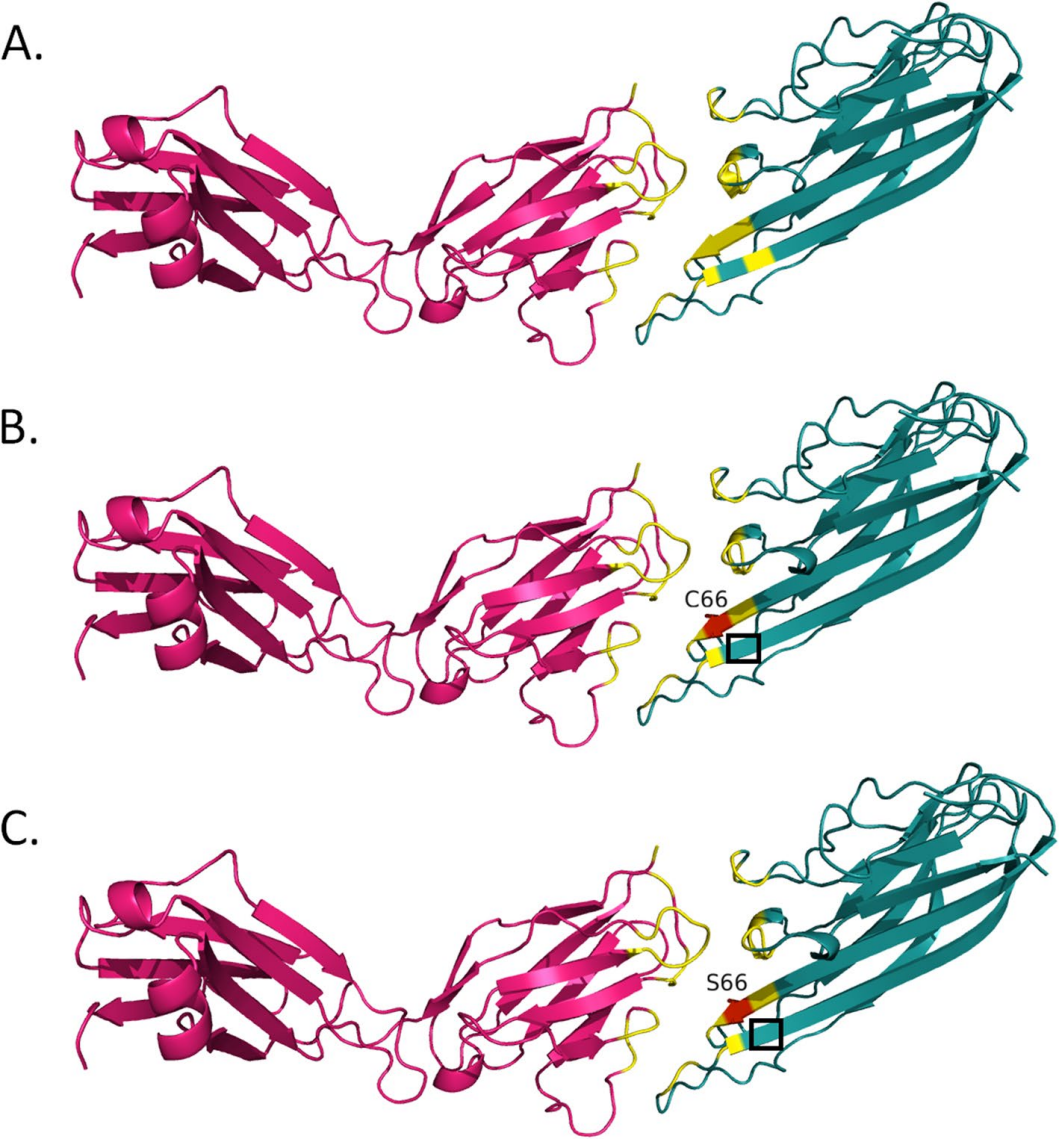
Interactions between TNFA and adalimumab. Interactions between the light chain of adalimumab and TNFA^WT^ (**A**), TNFA^G66C^ (**B**), and TNFA^G66S^ (**C**) structures. Hot pink- adalimumab light chain; deep teal- TNFA; yellow- interface; red- mutated residue. Regions with altered antigen-antibody interactions are denoted with black rectangles

In TNFA^WT^, the Glu23 residue was not in contact (cut-off value 4) with Asp1 residue in the L chain of ADA (Fig. 1A). The distance between Glu23 in TNFA (OE1) and Asp1 residue in the L chain was 4.065 Å (Fig. 1D). But both TNFA^G66C^ and TNFA^G66S^ variants changed the conformation and brought those two residues in contact with one another by reducing the distance to < 3.9 Å (Fig. 1B, 1C, 1E and 1F). TNFA^G66C^ and TNFA^G66S^ variants also caused structural changes in TNFA by expanding the cavity (surface pocket) by > 70 Å^3^ (Table 6). Furthermore, these two variants increased the buried surface area between TNFA and the L chain of ADA by > 100 Å^2^ compared to the wild-type complex, although the rest of the surface area of these two chains remained similar (Table 6). Additionally, alterations in the antigen-antibody interface region were observed in the case of these two variants (Fig. 2). These structural changes affected interactions between TNFA and the L chain of ADA mainly through the loss or weakening of van der Waals interactions and H-bonds.

**Table 6:** Effects of selected variants on TNFA structure and TNFA-mAb interactions

mAb	TNFA variants	TNFA structural changes	Ab chain	Surface area (Å^**2**^)		No. of bonds/interactions			
				Total	Buried	van der Waals	H-bonds	Weak H-bonds	Ionic interactions
ADA	WT	–	L	18368.58	1904.76	23	27	28	5
	G66C	Expansion of cavity volume by 74.088 Å^3^	L	18382.12	2044.42	21	23	31	5
	G66S	Expansion of cavity volume by 79.92 Å^3^	L	18380.93	2040.04	22	23	31	5
CZP	WT	–	H	19609.53	1285.39	32	33	38	5
	WT	–	L	18577.46	1819.46	34	36	36	9
	R131Q	No structural damage	H	19622.71	1284.04	33	32	38	5
	E135G	No structural damage	L	19622.71	1800.98	35	36	36	9
	R138Q	No structural damage	L	18570.75	1786.87	34	36	35	9
	R138W	No structural damage	L	18546.29	1841	34	36	36	9
GLM	WT	–	H	14262.13	2020.62	21	29	25	10
	Y141S	No structural damage	H	14314.38	2018.65	18	29	25	10
IFX	WT	–	L	18788.1	1193.27	25	28	39	9
	R138W	No structural damage	L	18802.43	1180.42	25	28	37	9

**Fig. 3:**
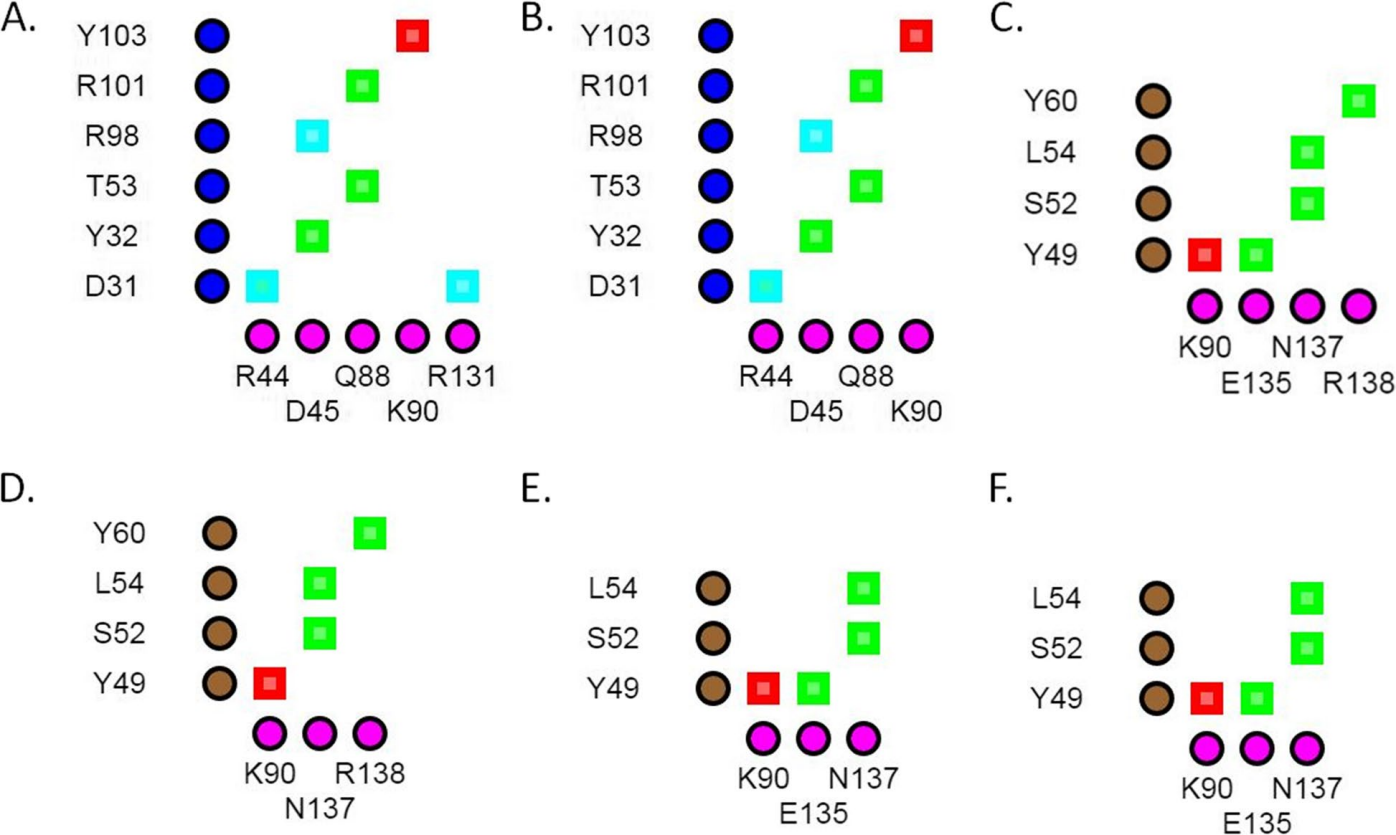
Interactions between TNFA and certolizumab pegol. 2D plots of interactions between TNFA and the heavy chain of certolizumab pegol in the wild-type (**A**) and TNFA^R131Q^ variant (**B**) structures. 2D plots of interactions between TNFA and the light chain of certolizumab pegol in the wild-type (**C**), TNFA^E135G^ (**D**), TNFA^R138Q^ (**E**), and TNFA^R138W^ (**F**) variant structures. TNFA residues are shown along the horizontal axis and certolizumab pegol residues are delineated along the vertical axis. Green- hydrogen bonds; cyan- salt bridges/ionic interactions; and red- cation-pi interactions

**Fig. 4:**
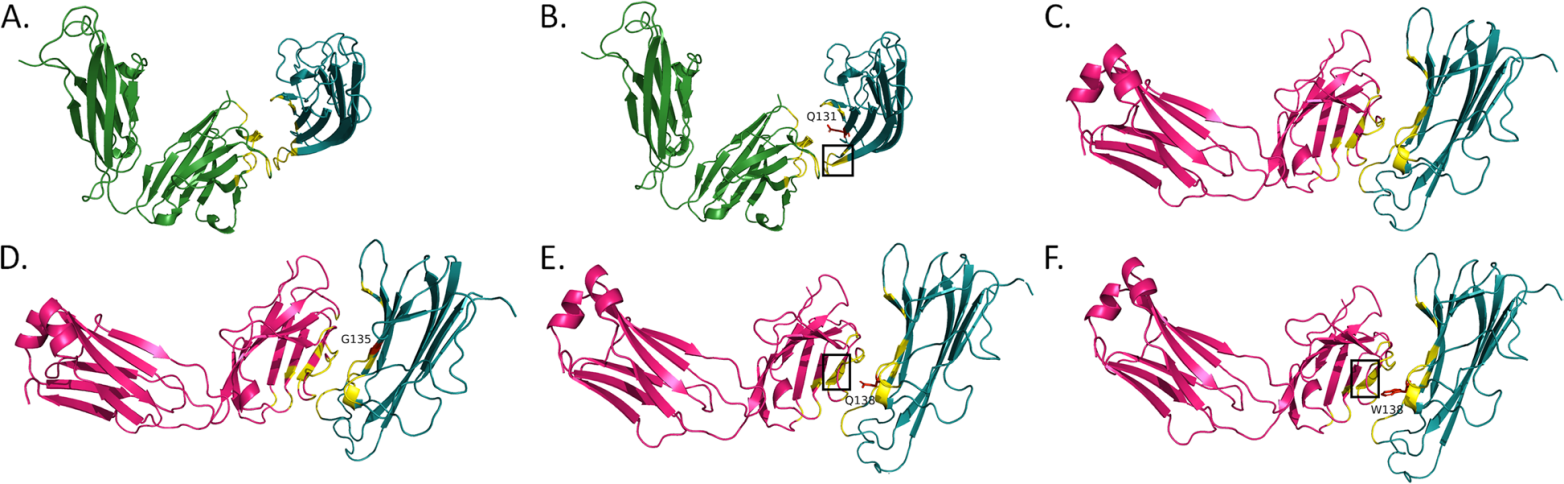
Interactions between TNFA and certolizumab pegol. Interactions between TNFA and the heavy chain of certolizumab pegol in the wild-type (**A**) and TNFA^R131Q^ variant (**B**) structures. Interactions between TNFA and the light chain of certolizumab pegol in the wild-type (**C**), TNFA^E135G^ (**D**), TNFA^R138Q^ (**E**), and TNFA^R138W^ (**F**) variant structures. Forest green- heavy chain of certolizumab pegol; hot pink-, light chain of certolizumab pegol; deep teal- TNFA; yellow- interface; red- mutated residue. Regions with altered antigen-antibody interactions are denoted with black rectangles

Change in TNFA structure or interaction at the TNFA-CZP interface was not caused by any of the four variants (TNFA^R131Q^, TNFA^E135G^, TNFA^R138Q^, and TNFA^R138W^) that were predicted by mCSM-PPI2, SAAMBE-3D, and MutaBind to destabilize the complex (Table 6). The ionic interaction between Arg131 in TNFA and Asp31 in the H chain of CZP was lost due to TNFA^R131Q^ (Fig. 3A and B). This loss of interaction caused structural changes in the antigen-antibody interface region (Fig. 4B). The H-bond between Glu135 of TNFA and Tyr49 in the L chain of CZP was lost in the presence of TNFA^E135G^ (Fig. 3C and D). Both TNFA^R138Q^ and TNFA^R138W^ caused the loss of H-bond between Arg138 of TNFA and Tyr60 in the L chain of CZP (Fig. 3E and F). These H-bond losses changed patterns of interacting residues in the CZP L chain (Fig. 4E and F).

**Fig. 5:**
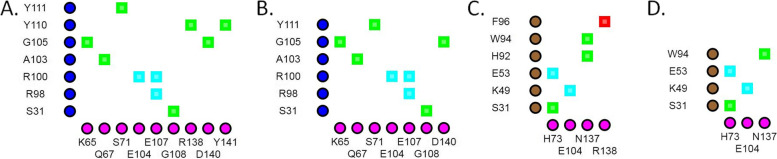
Interactions of TNFA with golimumab and infliximab. 2D plots of interactions between TNFA and the heavy chain of golimumab in the wild-type (**A**) and TNFA^Y141S^ variant (**B**) structures. 2D plots of interactions between TNFA and the light chain of infliximab in the wild-type (**C**) and TNFA^R138W^ variant (**D**) structures. TNFA residues are shown along the horizontal axis and antibody residues are delineated along the vertical axis. Green- hydrogen bonds; cyan- salt bridges/ionic interactions; and red- cation-pi interactions

**Fig. 6:**
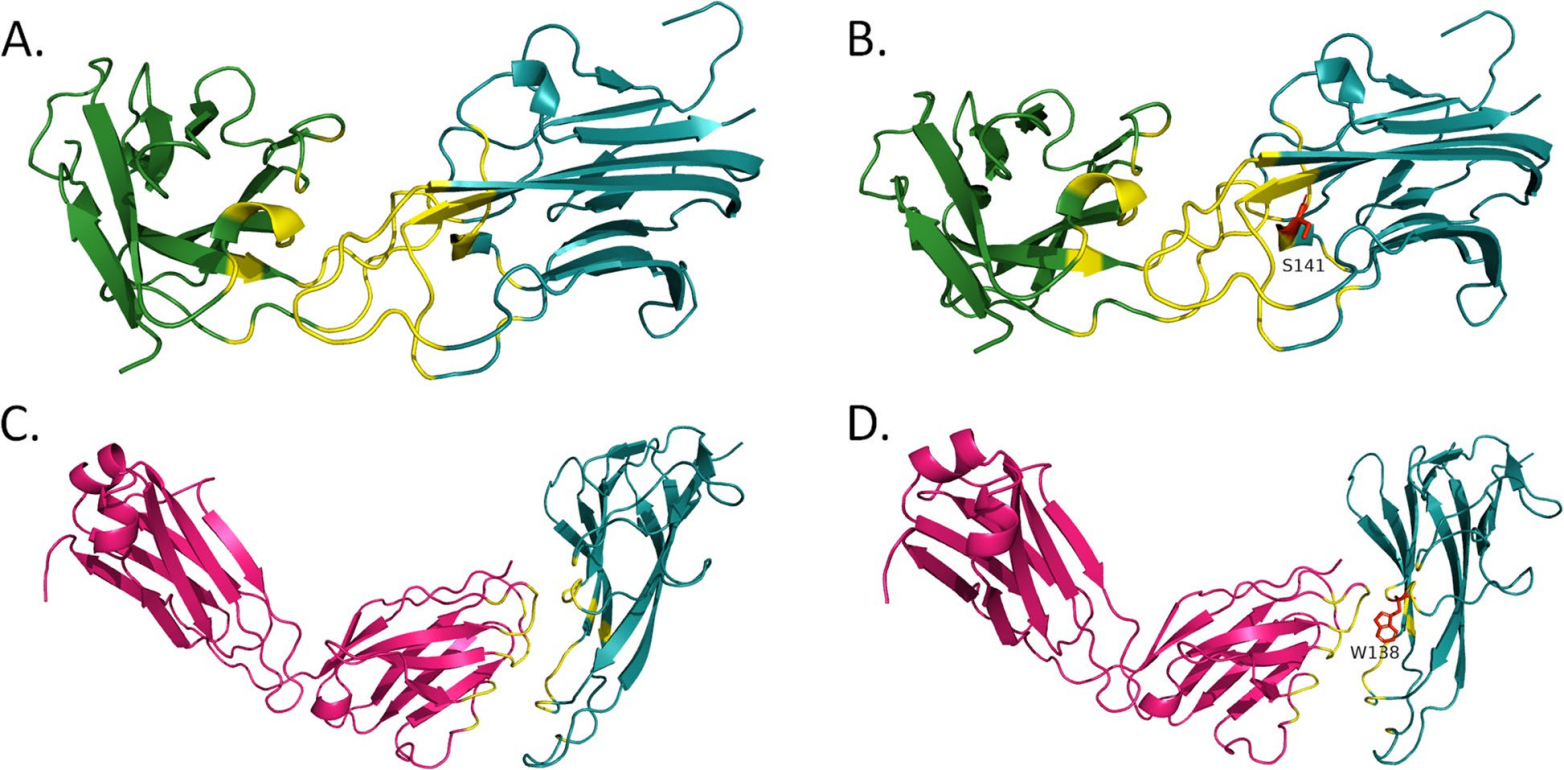
Interactions of TNFA with golimumab and infliximab. Interactions between TNFA and the heavy chain of golimumab in the wild-type (**A**) and TNFA^Y141S^ variant (**B**) structures. Interactions between TNFA and the light chain of infliximab in the wild-type (**C**) and TNFA^R138W^ variant (**D**) structures. Forest green- antibody heavy chain; hot pink- antibody light chain; deep teal- TNFA; yellow- interface; red- mutated residue

None of the TNFA^Y141S^ and TNFA^R138W^ variants caused any changes in TNFA structure or the interfaces of TNFA-GLM H chain and TNFA-IFX L chain, respectively (Table 6). TNFA^Y141S^ caused the loss of the H-bond between Tyr141 of TNFA and Tyr111 in the H chain of GLM (Fig. 5A and B). TNFA^R138W^ caused the loss of cation-pi interaction between Arg138 of TNFA and Phe96 in the L chain of IFX (Fig. 5C and D). These alterations however did not cause any noticeable changes at antigen-antibody interfaces (Fig. 6).

None of the seven variants (TNFA^G66C^, TNFA^G66S^, TNFA^R131Q^, TNFA^E135G^, TNFA^R138Q^, TNFA^R138W^, and TNFA^Y141S^), that were predicted to have substantial destabilizing effects on TNFA-mAb interactions, appeared to significantly destabilize TNFA-TNFR2 interactions unanimously by all the tools used in the analysis (Supplementary Table 1).

### Pathogenicity of the variants

Based on the data retrieved from DisGeNET and PhenoScanner databases, rs1454071630 (TNFA^G66C^ and TNFA^G66S^) is associated with generalized pustular psoriasis [51]. No other variant among the seven selected ones has been associated with any disease yet. But along with TNFA^G66C^ and TNFA^G66S^, TNFA^E135G^ was also predicted to have deleterious effects by all of the predictive tools (Table 7). Multiple tools predicted the TNFA^R138W^ and TNFA^Y141S^ variants to be disease-causing, whereas all tools used in this study predicted TNFA^R131Q^ and TNFA^R138Q^ to be non-pathogenic.

**Table 7:** Predicted pathogenicity of the selected variants

Variants	SIFT_Class	Polyphen_Class	PMut	Meta-SNP	PredictSNP1.0
TNFA^G66C^	Deleterious	Probably damaging	Disease	Disease	Deleterious
TNFA^G66S^	Deleterious	Possibly damaging	Disease	Disease	Deleterious
TNFA^R131Q^	Tolerated	Benign	Neutral	Neutral	Neutral
TNFA^E135G^	Deleterious	Possibly damaging	Disease	Disease	Deleterious
TNFA^R138Q^	Tolerated	Benign	Neutral	Neutral	Neutral
TNFA^R138W^	Tolerated	Possibly damaging	Neutral	Neutral	Deleterious
TNFA^Y141S^	Deleterious	Benign	Disease	Neutral	Deleterious

## Discussion

The aim of this study was to investigate the effects of epitopic missense variants in TNFA on interaction with corresponding therapeutic anti-TNFA antibodies and identify the candidate variants that may be an important predictor of responsiveness to these antibodies. In order to do so, the effects of those variants on the binding affinity of ADA, CZP, GLM, and IFX to TNFA were predicted using mCSM-PPI2, SAAMBE-3D, and MutaBind. These tools use different methods to predict ∆∆G. mCSM-PPI2 is a machine learning method that utilizes graph-based structural signatures known as mCSM [32]. These signatures encode distance patterns between atoms and can represent the protein residue environment and are used to train predictive models [52]. SAAMBE-3D is a development of the SAAMBE method, which uses the molecular mechanics/Poisson Boltzmann surface area (MM/PBSA) and knowledge-based terms to predict ∆∆G and amino acid–specific dielectric constant to mimic mutation-induced conformational flexibility [33]. MutaBind method uses molecular mechanics force fields, statistical potentials, and fast side-chain optimization algorithms [34]. Tools that use different methods may yield conflicting results, but their combined usage can help clarify the impacts of mutations [53]. The predicted destabilizing effects in our study were also assessed using 3D models of TNFA-mAb complexes. Variants that were predicted to substantially destabilize the TNFA-mAb interactions were selected for further analyses such as prediction of changes in TNFA structures and TNFA-mAb interfaces and calculation of van der Waals interactions, H-bonds, weak H-bonds, and ionic interactions because of their importance in antigen-antibody interactions [54]. Although none of these tools is supposed to be 100% accurate, we minimized the chances of erroneous predictions by using multiple tools and making consensus decisions. Direct experimental methods may provide more accurate results, but the use of in silicotools to prioritize the candidate variants may save time and effort [55].

The binding affinity of ADA may be affected by TNFA^G66C^ and TNFA^G66S^ variants (Table 2). Although Gly66 in TNFA does not directly interact with any residue of ADA, it is a part of the epitope [20]. Both TNFA^G66C^ and TNFA^G66S^ variants brought Glu23 of TNFA in contact with (within 4 Å) Asp1 of ADA in the L chain (Fig. 1C–F). Glu23 of TNFA contributes considerably to ADA binding [20]. Besides, two flanking residues of Gly66 in TNFA—Lys65 and Gln67—are essential for ADA binding [20]. Hence, the substitution of Gly66 in TNFA is likely to interfere with the binding affinity of ADA. Additionally, TNFA^G66C^ and TNFA^G66S^ significantly altered the TNFA structure in complex with ADA (cavity was expanded by > 70 Å^3^), but not in its trimeric state (PDB ID: 1TNF). Such changes could affect the stability of TNFA in complex with ADA [39]. These variants also increased the buried surface between TNFA and the L chain of ADA by > 100 Å^2^ and introduced observable changes in the antigen-antibody interface (Fig. 2B and C). Although the buried surface area between antigen and antibody may not reveal much information regarding the strength of their interactions [56], changes in such area may provide further evidence of structural alterations by TNFA^G66C^ and TNFA^G66S^. As a result of these alterations, there was weakening and/or losses of van der Waals interactions and H-bonds between TNFA^G66C^ and TNFA^G66S^ with the L chain of ADA.

Among the other epitopic missense variant positions, substitution of Glu135 in TNFA with an alanine caused dramatic reduction in TNFA-ADA affinity [20]. But such drastic reduction in presence of TNFA^E135G^ and TNFA^E135K^ variants was not observed, which might be due to interaction of ADA with Glu135 in an adjacent TNFA protomer [20].

TNFA^R131Q^, TNFA^E135G^, TNFA^R138Q^, and TNFA^R138W^ were predicted to affect TNFA-CZP interactions (Table 3). Substitution of Arg138 in TNFA with an Ala drastically decreases binding affinity to CZP [21]. In agreement with this finding, we found that both TNFA^R138Q^ and TNFA^R138W^ missense variants at this position may substantially reduce interaction with CZP. We also observed changes in the patterns of interacting residues in the CZP L chain in the case of these two variants (Fig. 4E and F). H-bond between Glu135 of TNFA and Tyr49 of CZP in the L chain plays a supplementary role in the energetics of TNFA-CZP interactions [21]. Although both TNFA^E135G^ and TNFA^E135K^ were predicted to destabilize the TNFA-CZP interactions with majority of the tools, these predictions could not be replicated with the 3D models of the TNFA^E135K^ variant. Substitution of both Arg138 and Glu135 in TNFA interrupted the formation of H-bonds between these residues and two Tyr residues of CZP (Tyr60 and Tyr49, respectively) in the L chain (Fig. 2C–E). Interactions between residues in epitopes and Tyr residues in paratopes are extremely important in the context of antigen-antibody affinity [57]. TNFA^R131A^ does not affect CZP binding affinity [21]. But in our study, TNFA^R131Q^ was predicted and determined to reduce the affinity of the H chain of CZP to TNFA in most of the cases as it caused the loss of salt bridge and/or ionic interaction between Arg131 of TNFA and Asp31 of CZP in the H chain (Fig. 3A and B). Side-chain atoms (NH1) of Arg131 in TNFA participate in this interaction (detected with iCn3D). So, the substitution of this residue is supposed to eliminate this salt bridge/ionic interaction with CZP, which may cause structural changes in the antigen-antibody interface (Fig. 4B).

In the present study, TNFA^Y141S^ was predicted to significantly destabilize (∆∆G > 1.0 kcal/mol) TNFA-GLM interactions by all the tools used (Table 4). This variant reduced the binding affinity of the H chain of GLM to TNFA. Tyr141 of TNFA forms the H-bond with Tyr110 of GLM in the H chain (Fig. 3A). TNFA^Y141S^ resulted in the loss of this H-bond (Fig. 3B). TNFA^R138W^ was predicted to drastically reduce (∆∆G > 1.0 Kcal/mol) interaction strength between TNFA and IFX by all predictive tools, but such reductions could not be detected in the 3D models of TNFA-IFX complex (Table 5). TNFA^R138W^ caused the loss of cation-pi interaction between Arg131 of TNFA and Phe96 of IFX in the L chain (Fig. 5C and D). Cation-pi interactions play a crucial role in stabilizing antigen-antibody complexes [58].

None of the variants, except TNFA^G66C^ and TNFA^G66S^, caused alteration in the TNFA structure in the complex with the corresponding mAbs or changed the buried surface area between TNFA and mAbs (Table 6). Besides, calculations by Arpeggio [41] hardly detected changes in the number of interactions in any other variant except TNFA^G66C^ and TNFA^G66S^. So, in the case of TNFA^G66C^ and TNFA^G66S^, structural alterations and eventual reductions in TNFA-ADA L chain interactions caused a decrease in TNFA-ADA affinity. In the case of the other three mAbs, the losses of some specific interactions (mostly H-bonds) caused reductions in the TNFA-mAb affinity. Another important finding of this study is that TNFA^R138W^ may interrupt both TNFA-CZP and TNFA-IFX interactions. So, individuals with this variant may be poor responders to both CZP and IFX. Further studies are needed to confirm this prediction. None of these variants affect the distribution of the TNFA residues that play important role in TNFA-TNFR2 interactions [20, 59]. So, these variants are less likely to affect interactions between TNFA and its receptor. This assumption was confirmed using predictive tools (Supplementary Table 1).

The seven missense variants (TNFA^G66C^, TNFA^G66S^, TNFA^R131Q^, TNFA^E135G^, TNFA^R138Q^, TNFA^R138W^, and TNFA^Y141S^) are present at low frequencies in the global population. TNFA^G66C^ and TNFA^G66S^, however, may be associated with autoimmune disease [51]. TNFA^E135G^ was predicted to be pathogenic by all the tools used in this analysis (Table 7). Among these tools, SIFT uses sequence homology to predict the effect of an amino acid substitution on protein function [60]. PolyPhen2 uses multiple protein sequence alignment pipeline and machine learning classification method to predict a missense variant’s pathogenicity [61]. PMut also uses the machine learning method [46]. Meta-SNP and Predict SNP 1.0 are consensus classifiers that utilize results from multiple high-performing classifier tools [47, 48]. So, it is highly probable that TNFA^E135G^ may be associated with disease. Besides, TNFA^R138W^ and TNFA^Y141S^ were predicted to be disease-causing by multiple tools. Further studies are needed to clarify if these three variants are associated with autoimmune diseases that are treated with anti-TNFA mAbs. If so, then at least these three variants along with TNFA^G66C^ and TNFA^G66S^ can be more frequent among patients who are treated with anti-TNFA mAbs compared with the rest of the population.

In order to contextualize our findings, we searched for ADA, CZP, GLM, and IFX response-associated SNPs in the Pharmacogenomics Knowledge Base (PharmGKB) [62]. We found twenty-one ADA, CZP, and IFX response-related SNPs. No GLM response-associated SNP was found. All these variant-drug combinations have level 3 clinical annotation,* i.e.*, evidence for those associations comes from single significant studies and the findings are yet to be replicated [62]. Variant alleles of these SNPs may be associated with poor [63] or better response [64]. The response may also depend on the disease being treated [65]. Only two (rs1799724 and rs361525) of these 21 SNPs were located in the *TNF* gene. rs1799724 and rs361525 are both located in the non-coding region and associated with responses to ADA [66] and IFX [67] in rheumatoid arthritis patients, respectively. So, there is a huge gap in our knowledge regarding the roles played by anti-TNFA mAb response-associated SNPs in their therapeutic outcome. Our study identifies seven missense variants in the epitopic region of TNFA, which may be predictive of the clinical outcomes of anti-TNFA mAb therapy regardless of the disease being treated. Moreover, this is the first study to our knowledge that has identified an association between a probable disease-causing variant (TNFA^Y141S^) and response to GLM. Therefore, our findings may contribute to the further clarification of the roles played by patients’ genotypes in their responses to ADA, CZP, GLM, and IFX.

## Conclusion

This study was inspired by the high rate (30–40%) of non-responsiveness to therapeutic anti-TNFA antibodies, which are used to treat multiple debilitating autoimmune diseases like rheumatoid arthritis, Crohn’s disease, ulcerative colitis, psoriasis, psoriatic arthritis, and ankylosing spondylitis. This in silico study predicts the candidate genetic variants that may underlie such variable responses to anti-TNFA antibodies. Based on these predictions, further studies may be designed and conducted using in vitro models to assess the effects of candidate variants on TNFA-mAb interactions. In addition, studies with patients undergoing treatments with anti-TNFA antibodies may shed more light on this heterogeneous responsiveness. Determining the genotypes at the candidate variant loci may improve clinical outcomes in patients. Also, population-specific studies might highlight the general genetic architecture at these loci, and thus help decide the more appropriate therapeutic antibodies for treatment.

## Supplementary Information

**Additional file 1: Supplementary Table 1.** Effects of missense variants on TNFA-TNFR2 interactions.

## Data Availability

All data generated or analyzed during this study are included in this published article (and its supplementary information files).

## Abbreviations

TNFA: Tumor necrosis factor alpha
mAb: Monoclonal antibody
ADA: Adalimumab
CZP: Certolizumab pegol
GLM: Golimumab
IFX: Infliximab
SNP: Single-nucleotide polymorphism
