# Supplementary material for: Missense variants in the TNFA epitopes and their effects on interaction with therapeutic antibodies—in silico analysis
Source: J Genet Eng Biotechnol. 2022 Jan 10;20:7. doi: 10.1186/s43141-021-00288-y (PMC8748575; doi:10.1186/s43141-021-00288-y)
Supplement: Supplementary file 1 — Additional file 1: Supplementary Table 1. Effects of missense variants on TNFA-TNFR2 interactions. [file 43141_2021_288_MOESM1_ESM.doc]

**Supplementary table 1:** Effects of missense variants on TNFA-TNFR2 interactions

| **Variants** | **∆∆G (kcal/mol)^a^** | | |
| --- | --- | --- | --- |
|  | mCSM-PPI2 | SAAMBE-3D | MutaBind2 |
| TNFA^G66C^ | 0.431 | 0.01 | 0.44 |
| TNFA^G66S^ | 0.143 | 0.09 | 0.6 |
| TNFA^R131Q^ | 0.046 | 1.12 | 0.02 |
| TNFA^E135G^ | 0.158 | 0.58 | 0.23 |
| TNFA^R138Q^ | -0.041 | 1.5 | 0.08 |
| TNFA^R138W^ | 0.094 | 0.99 | 0.17 |
| TNFA^Y141S^ | 0.355 | 0.92 | 0.39 |

^a^Positive values indicate decreasing affinity
